# Nosocomial Outbreak of Carbapenemase-Producing *Proteus mirabilis* With Two Novel *Salmonella* Genomic Island 1 Variants Carrying Different *bla*_NDM–1_ Gene Copies in China

**DOI:** 10.3389/fmicb.2021.800938

**Published:** 2022-01-13

**Authors:** Lang Yang, Hong He, Qichao Chen, Kaiying Wang, Yanfeng Lin, Peihan Li, Jinhui Li, Xiong Liu, Leili Jia, Hongbin Song, Peng Li

**Affiliations:** ^1^Chinese PLA Center for Disease Control and Prevention, Beijing, China; ^2^Department of Clinical Laboratory, People’s Liberation Army General Hospital Jingdong Medical District, Beijing, China; ^3^Academy of Military Medical Sciences, Academy of Military Sciences, Beijing, China

**Keywords:** *Proteus mirabilis*, *Salmonella* genomic island 1, clonal expansion, NDM-1, copy number variation

## Abstract

NDM-1-producing multidrug-resistant *Proteus mirabilis* brings formidable clinical challenges. We report a nosocomial outbreak of carbapenem-resistant *P. mirabilis* in China. Six *P. mirabilis* strains collected in the same ward showed close phylogenetic relatedness, indicating clonal expansion. Illumina and MinION sequencing revealed that three isolates harbored a novel *Salmonella* genomic island 1 carrying a *bla*_NDM–1_ gene (SGI1-1NDM), while three other isolates showed elevated carbapenem resistance and carried a similar SGI1 but with two *bla*_NDM–1_ gene copies (SGI1-2NDM). Four new single nucleotide mutations were present in the genomes of the two-*bla*_NDM–1_-harboring isolates, indicating later emergence of the SGI1-2NDM structure. Passage experiments indicated that both SGI variants were stably persistent in this clone without *bla*_NDM–1_ copy number changes. This study characterizes two novel *bla*_NDM–1_-harboring SGI1 variants in *P. mirabilis* and provides a new insight into resistance gene copy number variation in bacteria.

## Introduction

*Proteus mirabilis* has been recognized as one of the most common pathogens associated with nosocomial infections (Armbruster et al., 2017), particularly of the urinary and respiratory tracts, traumatic wounds, and surgical sites. *Salmonella* genomic island 1 (SGI1), a site-specific integrative mobilizable element initially found in *Salmonella enterica* serovar Typhimurium DT104 (Boyd et al., 2000), has emerged recently in *P. mirabilis* strains from diverse sources in China (Lei et al., 2014, 2015; Qin et al., 2015), France (Schultz et al., 2015; de Curraize et al., 2020), Egypt (Soliman et al., 2017, 2018) and South Korea (Sung et al., 2017). *Salmonella* genomic island 1 and its variants often carry numerous antimicrobial resistance genes and facilitate the emergence of multidrug resistant (MDR) *P. mirabilis*, thus posing a significant threat to public health.

New Delhi metallo-β-lactamase 1 (NDM-1), a mediator of carbapenem resistance, has become a serious challenge for clinical management since its first identification in 2009 (Yong et al., 2009). The *bla*_NDM–1_ gene is often located on plasmids and is rarely detected in an integrative element. To the best of our knowledge, there has been only one previous description of the integration of *bla*_NDM–1_ into an SGI1 relative structure in a *P. mirabilis* strain, which exhibited an unusual imipenem-resistant but meropenem-susceptible phenotype (Girlich et al., 2015). The clinical impact of *P. mirabilis* carrying *bla*_NDM–1_-positive SGI1 still requires further investigation.

We describe a nosocomial outbreak caused by a carbapenem-resistant *P. mirabilis* clone in China. Two novel SGI1 variants carrying one and two *bla*_NDM–1_ gene copies, respectively, were detected in six *P. mirabilis* strains through MiSeq and MinION sequencing. Phylogenetic analysis and passage experiments were further performed to gain an insight into the evolutionary relationship of both SGIs.

## Materials and Methods

### Bacterial Isolation and Antimicrobial Susceptibility Testing

From April to August in 2017, six *P. mirabilis* isolates were recovered from sputum or urine samples of hospitalized patients in the neurology department of the 263*^rd^* Hospital in Beijing, China. All isolates were collected through routine surveillance. Species-level identification and antimicrobial susceptibility were performed by a Vitek 2 compact system (bioMérieux, France). Results were interpreted according to the Clinical and Laboratory Standards Institute guidelines (Clinical and Laboratory Standards Institute, 2017). *Escherichia coli* ATCC25922 was used for quality control. Minimum inhibitory concentration (MIC) values of imipenem, meropenem and ertapenem were further determined by MIC test strips (Liofilchem, Italy). The entire *bla*_NDM–1_ gene was amplified with previously described primers and further confirmed by sequencing (Zou et al., 2015). The study was supervised by Ethics Committee of the Chinese PLA Center for Disease Control and Prevention. Informed consent was not required as no identifiable patient data was presented in this study.

### Transcriptional Expression Analysis of *bla*_NDM–1_ and Passage Experiments

The *bla*_NDM–1_ copy number in six strains were identified by Illumina MiSeq and Nanopore MinION sequencing (see below). Transcriptional expression of *bla*_NDM–1_ in PmBJ020-1 and PmBJ023-2 was determined by quantitative RT-PCR (qRT-PCR). Total RNA was isolated from bacterial cultures using RNApure Bacteria Kit (DNase I) (CWBio, China). RT-PCR was performed using UltraSYBR One Step RT-qPCR Kit (CWbio, China) in CFX96 Real-Time System (Bio-Rad, United States) as described previously (Wasfi et al., 2020). Expression levels were normalized relative to the transcriptional level of the constitutive 16S rRNA and interpreted by the ΔΔ*C*_*t*_ method (Johnson et al., 2013).

A passage experiment was performed on PmBJ020-1 and PmBJ023-2 with imipenem-containing (32 mg/L, Solarbio, China) and antibiotic-free broth (ThermoFisher, United States), respectively, for 12 h at 37°C. Cultures were adjusted to 0.5 McFarland with sterile PBS, 5 μl of which was added to 5ml of fresh broth. The OD600 of the overnight culture was measured using a Spectra Max M5 microplate reader (Molecular Devices, United States). This procedure was repeated for 15 consecutive days. Quantitative PCR (qPCR) was performed to quantify *bla*_NDM–1_ at the beginning and end of the passage experiment by QuantStudio3 (ThermoFisher, United States) as described previously (Wasfi et al., 2020). qPCR results were interpreted by the ΔΔ*C*_*t*_ method (Johnson et al., 2013). Quantitative RT-PCR was also performed to determine the transcriptional expression of *bla*_NDM–1_ after 30 passages. Statistical analysis was performed using the Student’s *t*-test to compare *bla*_NDM–1_ gene copy numbers and expression levels.

### Illumina/MinION Sequencing and Annotation

Total DNA was extracted from cultured bacteria using the High Pure PCR Template Preparation Kit (Roche). Whole genome sequencing (WGS) was conducted using an Illumina MiSeq platform with a 350-bp insert size. Genomic DNA was also sequenced using an Oxford Nanopore MinION sequencer with the RAD004 rapid sequencing kit. Sequencing was performed for 11 h on average for each sample. The *de novo* hybrid assembly of short Illumina reads and long MinION reads was performed using Unicycler (v0.4.0) with default parameters (Wick et al., 2017). Genome sequences were annotated using RAST (Aziz et al., 2008). Antimicrobial resistance genes were identified through alignment against the Comprehensive Antibiotic Resistance Database (Jia et al., 2017).

### Phylogenetic Analysis

Genome sequences of 75 available *P. mirabilis* isolates were downloaded from the NCBI database for phylogenetic analysis. *P. mirabilis* strain HI4320 (GenBank accession number AM942759.1) was used as the reference for alignment. Reads were mapped onto the reference genome using BWA (v0.7.12) (Li and Durbin, 2009). SNPs were identified using SAMtools (v1.3) (Li et al., 2009). A Maximum-Likelihood (ML) phylogenetic tree was constructed using RAxML (v8.2.4) with a general time reversible (GTR) model and a gamma distribution based on 1000 bootstraps (Stamatakis, 2014). Average nucleotide identities between genomes were calculated using JSpeciesWS to evaluate genome similarity (Richter et al., 2016).

### Nucleotide Sequence Accession Number

The genome sequences of *P. mirabilis* PmBJ004-1, PmBJ012-2, PmBJ015-2, PmBJ020-1, PmBJ023-2 and PmBJ024-1 have been deposited in GenBank under accession numbers JADOZD000000000, CP065148, CP065147, CP065146, CP065145 and CP065144, respectively.

## Results

### Clonal Expansion of *Proteus mirabilis* in the Same Ward

**FIGURE 1:**
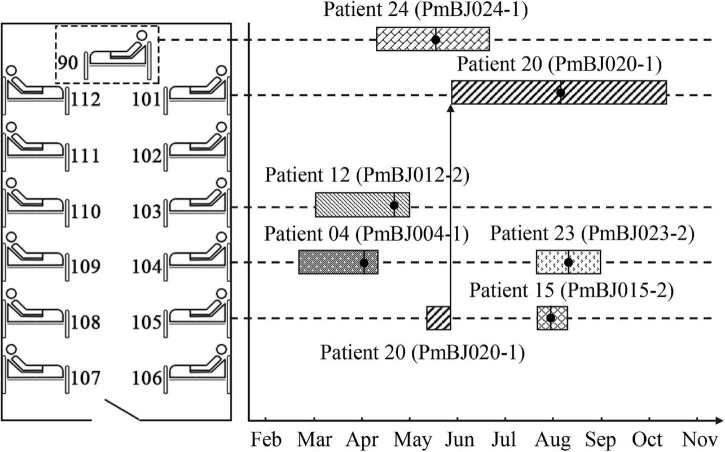
Spatial and temporal distribution of patients during hospitalization. Bed 90 was temporarily placed in the neurology ward; the dotted box does not reflect its exact position. The boxes represent the stay of the patients. The vertical bars with black dots indicate the isolation date of *P. mirabilis*.

In 2017, six *P. mirabilis* isolates (designated PmBJ004-1, PmBJ012-2, PmBJ015-2, PmBJ020-1, PmBJ023-2 and PmBJ024-1, respectively) were recovered. The first isolation was from the sputum of a patient in the neurology department diagnosed with cerebral infarction and coronary artery disease. During the following four months, five new cases were identified in the same ward. Four isolates were recovered from the sputum cultures and 1 from the urine culture. Five of the six patients had stayed in three adjacent beds, one of which moved to a separate bed afterward (Figure 1). Patient 24 had lain on a temporarily placed bed.

**FIGURE 2:**
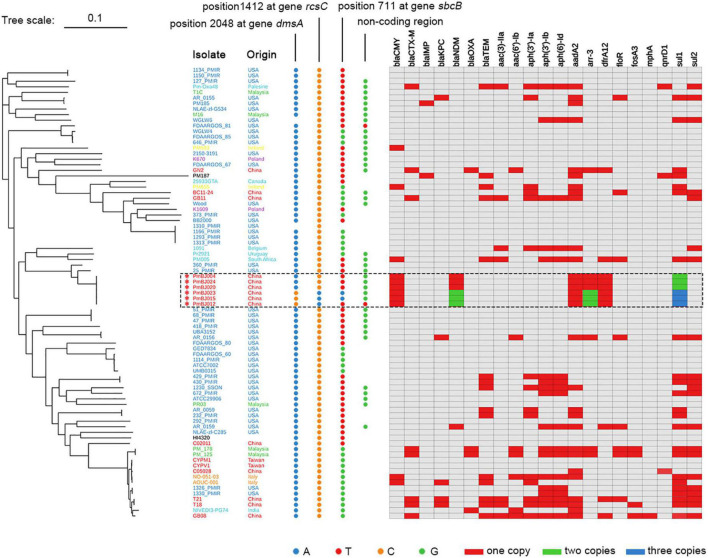
ML Phylogenetic tree of *P. mirabilis* isolates with key SNPs and resistance gene profiles. The six *P. mirabilis* strains are marked with an asterisk. Nucleotides at four loci that distinguish the six isolates are indicated by dots in different colors. The colored blocks indicate resistance gene copy numbers.

The phylogenetic tree was constructed based on genomic sequences of the six *P. mirabilis* isolates by short-read and long-read WGS and 75 available *P. mirabilis* complete genomes in NCBI (Figure 2). Topology revealed that the six *P. mirabilis* isolates were located within one clade. They exhibited very high average nucleotide identities ranging from 99.93 to 99.99%, indicating a clonal expansion. The six *P. mirabilis* isolates were distinct from other Chinese strains in the phylogeny but showed closer relationship with overseas strains 360_PMIR and 25_PMIR from the United States, indicating a different evolutionary origin.

Further analysis revealed that isolates PmBJ012-2, PmBJ015-2, and PmBJ023-2 were distinguished from PmBJ004-1, PmBJ020-1, and PmBJ024-1 by four single nucleotide mutations (Figure 2). One was located within the *dmsA* gene associated with dimethyl sulfoxide reduction and resulted in the amino acid substitution of Glu683Gly, and the other was a synonymous mutation at position 1412 (C to T) of the capsular synthesis gene *rcsC*. PmBJ015-2 and PmBJ023-2 had a synonymous mutation at position 711 (A to T) of exodeoxyribonuclease I gene *sbcB*, and PmBJ012-2 had a unique nucleotide substitution (G to A) in a non-coding region.

### Genetic Features of Two Novel *Salmonella* Genomic Island 1 Variants Carrying One and Two *bla*_NDM–1_ Gene Copies

**FIGURE 3:**
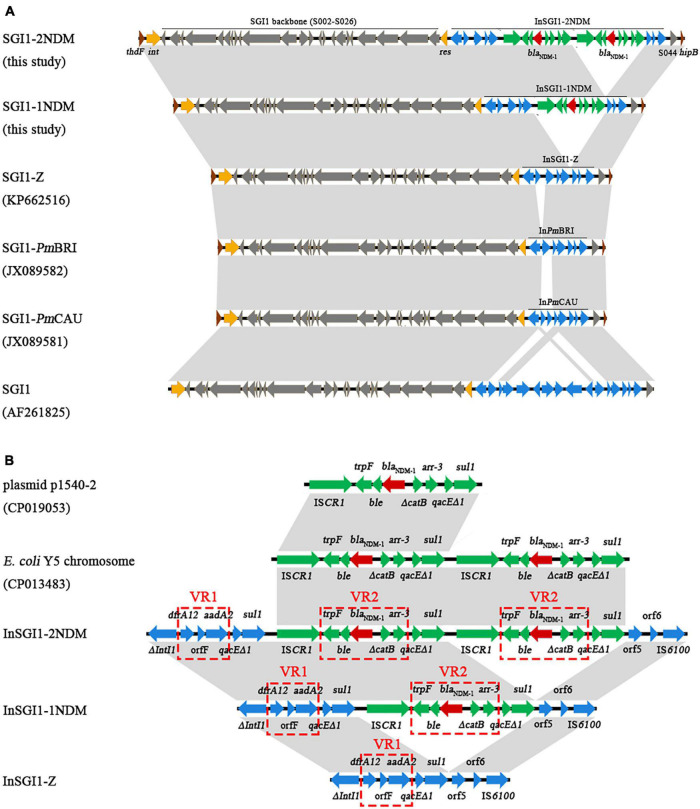
Comparative schematic diagram of **(A)** SGI1s and **(B)** the MDR regions of SGI1s. Arrows represent the positions and directions of gene transcription. The red, green and blue arrows indicate genes in the MDR regions. The gray-shaded areas represent regions sharing >99% identity.

Sequence analysis revealed that the six *P. mirabilis* isolates have an SGI1 integrated between the *thdF* and *hipB* chromosomal genes. 18-bp direct repeats (DRs) of specific recombination sites *attB* (DR-R) and *attP* (DR-L) were identified. Further alignment against existing SGI1s indicated that PmBJ004-1, PmBJ020-1, and PmBJ024-1 harbored a novel SGI1 structure designated SGI1-1NDM; while PmBJ012-2, PmBJ015-2, and PmBJ023-2 carried a different SGI1 variant named SGI1-2NDM (Figure 3A). SGI1-1NDM had a length of 40,293bp, while SGI1-2NDM contained 46,693bp. The two SGI1s shared a backbone of 27.4kb that was highly identical to that of SGI1 from *S.* Typhimurium DT104 (Boyd et al., 2001). The MDR regions of SGI1-1NDM and SGI1-2NDM were integrated between the backbone genes *res* (S027) and S044, at the same position as in the *Salmonella* SGI1.

The MDR region (InSGI1-1NDM) of SGI1-1NDM harbored a complex class 1 integron of the In4 type (Figure 3B). 5-bp DRs, ACTTG, created upon transposition were detected. This integron was organized as 5′CS (*intI1*), variable region 1 (VR1), 3′CS-1 (*qacE*Δ*1*/*sul1*), IS*CR1*, VR2 and 3′CS-2 (*qacE*Δ*1*/*sul1*). The VR1 carried a *dfrA12-orfF-aadA2* cassette array conferring resistance to trimethoprim, spectinomycin and streptomycin, which has been found in the class 1 integron of a genomic island SGI1-Z (KP662516.1) in *P. mirabilis* PM58 (Qin et al., 2015). The VR2 comprised a typical *bla*_NDM–1_ unit (*trpF-ble-bla*_NDM–1_), a truncated *catB*, and an *arr-3* gene. This VR2 structure flanked by IS*CR1* and 3′CS has also been found in several enterobacterial plasmids including *E. coli* plasmids p1540-2 (CP019053.1), p6061604-KPC (MN823987.1), and pHNSD133T1 (MG196293.1) (Lv et al., 2018), *Citrobacter freundii* plasmid pCf75 (CP047308.1) (Li et al., 2020), and *Klebsiella pneumoniae* plasmid pNDM_3214 (CP028851.1) (Liu and Su, 2019).

The MDR region (InSGI1-2NDM) of SGI1-2NDM also contained an In4-type complex class 1 integron. Compared with InSGI1-1NDM, the InSGI1-2NDM has acquired a second *bla*_NDM–1_-carrying VR2 together with adjacent IS*CR1* and 3′CS. Thus, the InSGI1-2NDM could be divided into 5′CS, VR1, 3′CS-1, IS*CR1*, VR2, 3′CS-2, IS*CR1*, VR2, and 3′CS-3. The two VR2s presented 100% nucleotide identity, and both IS*CR1* elements had *ori*IS sites detected downstream. This structure with two tandem *bla*_NDM–1_ copies was similar to that in the *E. coli* Y5 chromosome, whereas the latter had a different VR1 carrying *aac(6*′*)lb-cr*, *arr-3*, and *aadA16*.

### Higher Carbapenem Resistance and *bla*_NDM–1_ Expression Level of SGI1-2NDM Relative to SGI1-1NDM

**FIGURE 4:**
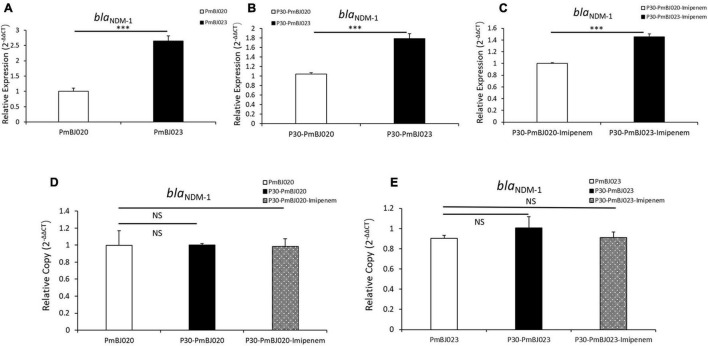
Expression level and copy number of the *bla*_*NDM–1*_ gene in PmBJ020 and PmBJ023 during passage experiment. Relative expression of *bla*_*NDM–1*_ in PmBJ020 and PmBJ023 **(A)**, P30-PmBJ020 and P30-PmBJ023 **(B)**, P30-PmBJ020-Imipenem and P30-PmBJ023-Imipenem **(C)**. Copy number of *bla*_*NDM–1*_ in PmBJ020, P30-PmBJ020 and P30-PmBJ020-Imipenem **(D)**, PmBJ023, P30-PmBJ023 and P30-PmBJ023-Imipenem **(E)**. P30-PmBJ020, PmBJ020 after 30 passages without antibiotic selection; P30-PmBJ023, PmBJ023 after 30 passages without antibiotic selection; P30-PmBJ020-Imipenem, PmBJ020 after 30 passages with imipenem selection; P30-PmBJ023-Imipenem, PmBJ023 after 30 passages with imipenem selection. Significance of difference was determined using the Student’s *t*-test (*** *P* < 0.001; NS, not significant).

All six *P. mirabilis* isolates were resistant to ampicillin, ciprofloxacin, nitrofurantoin, levofloxacin and most cephalosporins but susceptible to amikacin, aztreonam, gentamicin, and tobramycin (Table 1). MIC test strips showed that all the isolates were resistant to imipenem, but the imipenem MICs of PmBJ012-2, PmBJ015-2 and PmBJ023-2 carrying SGI1-2NDM (96 μg/ml, 96 μg/ml and 64 μg/ml, respectively) were remarkably higher than those of PmBJ004-1, PmBJ020-1 and PmBJ024-1 carrying SGI1-1NDM (16 μg/ml, 16 μg/ml and 12 μg/ml, respectively). MIC test strips also revealed that PmBJ012-2, PmBJ015-2 and PmBJ023-2 were resistant to meropenem and ertapenem, while PmBJ004-1, PmBJ020-1 and PmBJ024-1 were intermediate to meropenem and susceptible to ertapenem. qRT-PCR revealed that the expression level of *bla*_NDM–1_ in PmBJ023-2 was 2.63-fold higher than that in PmBJ020-1 (Figure 4A).

**TABLE 1:** Clinical details and antibiotic susceptibilities of six *P. mirabilis* isolates.

Characteristic	PmBJ004-1	PmBJ012-2	PmBJ015-2	PmBJ020-1	PmBJ023-2	PmBJ024-1
Patient	04	12	15	20	23	24
Specimens	Sputum	Sputum	Sputum	Sputum	Sputum	Urine
Admission date	February 19	March 1	July 21	May 12	July 19	April 8
Discharge date	April 11	April 29	August 9	October 8	August 30	June 20
Isolation date	April 1	April 21	July 30	August 04	August 11	May 18
Antibiotic susceptibilities determined by Vitek 2 (MIC, μg/ml)						
Amikacin	≤ 2 (S)	4 (S)	4 (S)	≤ 2 (S)	≤2 (S)	≤ 2 (S)
Ampicillin	≥ 32 (R)	≥32(R)	≥ 32 (R)	≥32(R)	≥ 32 (R)	≥32(R)
Ampicillin-sulbactam	≥ 32 (R)	≥32(R)	≥ 32 (R)	≥32(R)	≥ 32 (R)	≥32(R)
Aztreonam	≤ 1 (S)	≤1 (S)	≤ 1 (S)	≤1 (S)	≤ 1 (S)	≤1 (S)
Cefazolin	≥ 64 (R)	≥64(R)	≥ 64 (R)	≥64(R)	≥ 64 (R)	≥64(R)
Ceftazidime	≥ 64 (R)	≥64(R)	≥ 64 (R)	≥64(R)	≥ 64 (R)	≥64(R)
Ceftriaxone	16 (R)	32 (R)	32 (R)	32 (R)	32 (R)	16 (R)
Cefuroxime Axetil	≥ 64 (R)	≥64(R)	≥ 64 (R)	≥64(R)	≥ 64 (R)	≥64(R)
Cefuroxime Sodium	≥ 64 (R)	≥64(R)	≥ 64 (R)	≥64(R)	≥ 64 (R)	≥64(R)
Ciprofloxacin	≥ 4 (R)	≥4 (R)	≥ 4 (R)	≥4 (R)	≥ 4 (R)	≥4 (R)
Gentamicin	≤ 1 (S)	≤1 (S)	≤ 1 (S)	≤1 (S)	≤ 1 (S)	≤1 (S)
Levofloxacin	≥ 8 (R)	≥8 (R)	≥ 8 (R)	≥8 (R)	≥ 8 (R)	≥8 (R)
Nitrofurantoin	128 (R)	128 (R)	128 (R)	128 (R)	256 (R)	128 (R)
Piperacillin	32 (I)	32 (I)	32 (I)	32 (I)	32 (I)	32 (I)
Tobramycin	≤ 1 (S)	≤1 (S)	≤ 1 (S)	≤1 (S)	≤ 1 (S)	≤1 (S)
Trimethoprim-Sulfamethoxazole	≤ 20 (S)	≥ 320 (R)	≥320 (R)	≤ 20 (S)	≥ 320 (R)	≤ 20 (S)
Antibiotic susceptibilities determined by MIC test strips (MIC, μg/ml)						
Ertapenem	0.5 (S)	12 (R)	16 (R)	0.38 (S)	32 (R)	0.19 (S)
Imipenem	16 (R)	96 (R)	96 (R)	16 (R)	64 (R)	12 (R)
Meropenem	2 (I)	24 (R)	24 (R)	2 (I)	64 (R)	1.5 (I)

### Stable Persistence of Novel *Salmonella* Genomic Island 1 Structures

We assessed the stability of the two SGI1s by passage experiments with and without antibiotic selection. qPCR revealed that the *bla*_NDM–1_ copy number remained unchanged in both SGI1s during passage experiments (Figures 4D,E). Quantitative RT-PCR indicated that the expression level of *bla*_NDM–1_ in PmBJ023-2 was still 1.46- and 1.72-fold higher than that PmBJ020-1 with and without imipenem selection, respectively, after 30 passages (Figures 4B,C). These results indicated that the two novel SGI1s had strong structural and functional stability and persisted in *P. mirabilis*.

## Discussion

This study documents a nosocomial outbreak caused by six carbapenem-resistant *P. mirabilis* strains in a Chinese hospital. To the best of our knowledge, this is also the first report of a hospital outbreak of an NDM-1-producing *P. mirabilis* clone. Since environmental samples were not collected in this ward, the transmission route of the *P. mirabilis* clone was unidentifiable. Additional cases were identified during the following four months in the same ward, suggesting long term clonal colonization. Infections caused by such MDR pathogens may result in increased morbidity and mortality due to the inefficacy of commonly used antibiotics. To prevent further spread of such pathogens, sterilization of multi-use instruments and caring devices should be vigorously implemented. Contact precautions should be taken with improved hand-hygiene compliance and glove use.

The presence of similar components indicated a close relation of SGI1-1NDM and SGI1-2NDM to SGI1-Z. However, the DR-R diverged in 1bp from the DR-L in SGI1-Z, which was not found in two SGI1 variants in this study. It is likely that SGI1-1NDM or SGI1-2NDM were not derived from SGI1-Z, but from another similar structure. IS*CRI1* serves as a common vehicle for the mobilization of antibiotic resistance genes by rolling circle transposition (Janvier et al., 2013; Chen et al., 2014; Li et al., 2014). A previous study demonstrated that the *bla*_NDM–1_ gene could be transferred via a circular IS*CR1-bla*_NDM–1_ element released from plasmids (Li et al., 2014). Since the *ori*IS site was detected, we speculate that the IS*CR1* element might mediate the acquisition of the *bla*_NDM–1_-embedded VR2 to an SGI1-Z-like structure, presumably through recombination at the *sul1* locus of the class 1 integron, to form the novel variant SGI1-1NDM.

Although two tandem copies of the *bla*_NDM–1_ gene have been found in several bacterial isolates (Jovcić et al., 2013; Shen et al., 2017; Feng et al., 2018), dynamic *bla*_NDM–1_ copy number variations during clonal expansion have been detected very rarely under natural conditions. In this study, PmBJ012-2, PmBJ015-2, and PmBJ023-2 with two *bla*_NDM–1_ copies were not distinguished from PmBJ004-1, PmBJ020-1, and PmBJ024-1 based on clinical information such as isolation date or source. We also did not find conversion between SGI1-1NDM and SGI1-2NDM during passage experiments. However, four additional nucleotide mutations at the genome-wide level were found in PmBJ012-2, PmBJ015-2, and PmBJ023-2. This observation strongly suggests that the isolates with two *bla*_NDM–1_ copies might have evolved later than those with single copy. It is likely that SGI1-1NDM with a single *bla*_NDM–1_ copy was the ancestral structure and preceded a second similar transposition event that resulted in the formation of SGI1-2NDM with two copies. The findings of this study also provided evidence that two tandem *bla*_NDM–1_ copies were not transferred as a block, but formed due to a multiplication mechanism. Although the strains harboring two *bla*_NDM–1_ gene copies showed elevated carbapenem resistance, it was still not clear whether antimicrobial pressure played a crucial role in the emergence of SGI1-2NDM. The strong structural stability of both SGI1s even under antibiotic-free conditions suggest their high potential for long-term persistence, which warrants further studies.

## Conclusion

This study describes a nosocomial outbreak caused by carbapenem-resistant *P. mirabilis* in China. Six *P. mirabilis* strains collected in the same ward were closely related, indicating clonal expansion. Illumina and MinION sequencing revealed the presence of a novel SGI1-1NDM carrying one *bla*_NDM–1_ gene copy and an SGI1-2NDM carrying two *bla*_NDM–1_ gene copies. Four new single nucleotide mutations in the genomes of the SGI1-2NDM-harboring isolates indicate the possible later emergence of SGI1-2NDM. SGI1-2NDM confers higher carbapenem resistance and *bla*_NDM–1_ expression level relative to SGI1-1NDM. Both SGIs were stably persistent with and without antibiotic selection. Vigorous surveillance and urgent actions should be taken immediately to control the continuous expansion of such pathogens.

## Data Availability Statement

The datasets presented in this study can be found in online repositories. The names of the repository/repositories and accession number(s) can be found below: https://www.ncbi.nlm.nih.gov/genbank/, JADOZD000000000; https://www.ncbi.nlm.nih.gov/genbank/, CP065148; https://www.ncbi.nlm.nih.gov/genbank/, CP065147; https://www.ncbi.nlm.nih.gov/genbank/, CP065146; https://www.ncbi.nlm.nih.gov/genbank/, CP065145; and https://www.ncbi.nlm.nih.gov/genbank/, CP065144.

## Author Contributions

PnL and LY performed data analysis and prepared the manuscript. HH and QC collected samples and performed susceptibility testing. JL, YL, and KW performed DNA extraction and genome sequencing. PiL, XL, and LJ performed genome assembly and annotation. PnL and HS designed the study and revised the manuscript. All authors contributed to review and revision, and approved the final version.

## Conflict of Interest

The authors declare that the research was conducted in the absence of any commercial or financial relationships that could be construed as a potential conflict of interest.

## Publisher’s Note

All claims expressed in this article are solely those of the authors and do not necessarily represent those of their affiliated organizations, or those of the publisher, the editors and the reviewers. Any product that may be evaluated in this article, or claim that may be made by its manufacturer, is not guaranteed or endorsed by the publisher.
